# Impact of Clinician Experience on Intraosseous Anesthesia Outcomes in Endodontic Treatment: Retrospective Cohort Study

**DOI:** 10.3390/dj13060238

**Published:** 2025-05-27

**Authors:** Ahmed I. Alali, Jacqueline Lopez Gross, Mustafa Ayad Al-Sumaidaee, Michelle Wong, Sean Xinyang Feng, Pavel S. Cherkas, Bettina R. Basrani

**Affiliations:** 1 Faculty of Dentistry, University of Toronto, Toronto, ON M5G 1G6, Canada; ahm.ismail@mail.utoronto.ca (A.I.A.); michelle.wong@dentistry.utoronto.ca (M.W.); pavel.cherkas@dentistry.utoronto.ca (P.S.C.); 2 Private Practice, London, ON N6B 2P9, Canada; jlopezgross@gmail.com; 3 Private Practice, Baghdad 10011, Iraq; mustafa.alsumaidaee@utoronto.ca; 4 Dalla Lana School of Public Health, University of Toronto, Toronto, ON M5T 3M7, Canada; xinyang.feng@mail.utoronto.ca

**Keywords:** Clinician experience, endodontic resident, endodontist, intraosseous anesthesia, QuickSleeper 5, endodontic treatment, mandibular molars

## Abstract

**Objectives**: Although many studies have demonstrated the effectiveness of intraosseous anesthesia (IOA), the experience of clinicians administering IOA has rarely been reported. Some endodontists may never have received formal training in IOA, raising questions about whether inexperienced clinicians can achieve similar results. Although the previous studies suggest that limited experience may increase the risk of complications, the extent to which clinician experience influences IOA outcomes remains uncertain. This study sought to assess whether clinician experience affects the effectiveness, patient comfort, and safety of IOA in endodontic treatment. **Methods**: This retrospective cohort study included 72 patients who had previously undergone endodontic treatment or retreatment for mandibular molars. Of these, 37 were treated by an endodontic resident with limited IOA experience, and 35 by an endodontist with extensive IOA experience. IOA was administered using the QuickSleeper 5 system (Dental Hi Tec, Cholet Cedex, France) as the sole anesthetic technique before the start of the endodontic treatment or retreatment. Pain assessments were recorded preoperatively (baseline), during IOA administration, and during the treatment. Statistical analyses included binomial and chi-square tests to compare the success rates and associations between the clinician experience and the IOA outcomes. Statistical significance was set at *p* < 0.05. **Results**: Pain during IOA administration was reported as none or mild in 98.6% of the patients, with no significant difference between the endodontist (97.2%) and resident (100%) groups (*p* = 0.378). Profound anesthesia was achieved in 94.1% of the cases, with no significant difference between the endodontist (96.8%) and resident (91.9%) groups (*p* = 0.738). Complications were rare, with two cases of perforator separation (5.4%) in the resident group; nevertheless, clinician experience was not significantly associated with complications (*p* = 0.553). Conclusions: Within the limitations of this study, clinician experience did not significantly affect the IOA outcomes, including effectiveness, patient comfort, and safety. These findings suggest that IOA can be used effectively by clinicians with different levels of experience, thus supporting its broader implementation in endodontic practice.

## 1. Introduction

Profound local anesthesia is necessary for endodontic treatment and retreatment. The inferior alveolar nerve block (IANB) introduced by William Stewart Halsted in 1884 [1] is the most commonly used local anesthetic technique for mandibular molars [2,3]. However, its effectiveness is highly variable. The success rates for IANB in non-inflamed pulps have been reported to range from 42% to 73%, with individual studies demonstrating 42% in a sample of 40 patients [4] and 54% in a sample of 40 patients [5]. In cases of symptomatic irreversible pulpitis, the success rates decline to 19–56%, as reported in studies with success rates of 19% in a sample of 26 patients [6], 31% in a sample of 48 patients [7], and 56% in a sample of 61 patients [8].

Due to this inconsistency, various strategies have been explored to improve the efficacy of mandibular anesthesia. Some studies have investigated supplementary techniques to enhance IANB success, including periodontal ligament injection and buccal infiltration. Periodontal ligament injection administered through a computer-controlled local anesthetic delivery system has demonstrated moderate success, achieving pulpal anesthesia in 56% of cases when IANB alone was ineffective [9]. However, its duration is limited, with anesthesia persisting for only 23 min in the first molars [5].

Similarly, buccal infiltration using 4% articaine has been investigated as an adjunct to IANB. Although it has been found to improve the anesthetic success rates, its effectiveness remains inconsistent, with reported success ranging from 42% to 58% [10,11].

While these supplementary techniques have demonstrated varying degrees of effectiveness, some studies have proposed replacing IANB entirely with alternative primary techniques, such as the Gow-Gates mandibular nerve block introduced in 1973 [12] and the Vazirani–Akinosi [13] nerve block first introduced in 1960, and later reintroduced and refined in 1977. However, these techniques have not gained widespread adoption in dental practice, primarily because of their slower onset and lower success rates, particularly in cases involving symptomatic irreversible pulpitis [14,15].

Given these challenges, intraosseous anesthesia (IOA) has emerged as a promising alternative for achieving profound anesthesia in the mandibular molars. Originally introduced in 1910 [16], IOA has been used for decades, although the early techniques—relying on bone perforation with needles, burs, and reamers—were initially limited by infection risks before the advent of antibiotics [17]. A more modern approach to IOA was introduced nearly three decades ago with the Stabident system (Fairfax Dental, Inc., Miami, FL, USA) [4].

Initially, IOA was primarily used as a supplementary technique to enhance the effectiveness of other anesthetic methods [6,7,18,19]. However, more recent studies have explored its use as a primary technique for achieving anesthesia in mandibular molars requiring endodontic treatment or retreatment [20,21].

The IOA technique entails the direct delivery of anesthesia into the cancellous bone surrounding the target tooth [22]. IOA has the following advantages: quick onset [4,18,23,24,25,26], minimal or no pain during IOA administration in the majority of patients (85–100%) [4,6,19,23,26], and high success rates (81.2–96.8%) when used as the sole anesthetic technique for mandibular molars requiring endodontic treatment [20,21,26,27,28].

However, certain limitations have been reported, including transient increases in heart rate [4,6,19,26,29,30,31,32], a shorter duration of anesthesia [25,26,31,33], and postoperative soreness and hyperocclusion [4,34].

Although considered a safe technique, IOA has been associated with various complications, including swelling, purulence at the injection site, and perforator separation, with an incidence of less than 5% [4,28,33,35]. One case report described bone necrosis and the loss of two teeth, which was partially attributed to the clinician’s inexperience [36].

Despite their demonstrated effectiveness, the use of IOA in endodontic practice remains limited. A systematic review highlighted its limited clinical adoption, likely owing to concerns regarding potential complications [3]. Additionally, a survey of endodontists found that many clinicians do not use IOA, possibly owing to the perceived challenges in its administration or limited formal training in endodontic programs [37]. This limited clinical use persists despite well-documented inconsistencies in IANB effectiveness [4,5], suggesting that IOA could be an underutilized solution for achieving more predictable anesthesia for mandibular molars requiring endodontic treatment.

A critical gap in the literature is the lack of evidence on how clinician experience influences IOA administration and outcomes. While multiple studies have reported the success and safety of IOA, they do not specify the level of experience of the clinicians performing injections, leaving uncertainty regarding whether experience influences the effectiveness or complication rates. Unlike IANB, which is routinely taught in dental curricula, IOA may require a steeper learning curve owing to its technique-sensitive nature, potentially leading to outcome variability. One case report suggested that clinician inexperience may contribute to procedural difficulties and adverse events, such as perforation-related issues and localized tissue damage [36]; however, no study has directly examined this relationship.

Therefore, this study aimed to evaluate the impact of clinician experience on IOA outcomes, specifically by examining pain during administration, effectiveness in achieving profound anesthesia, and the incidence of complications.

By addressing this gap, this study provides an insight into whether clinician experience significantly affects the IOA outcomes and whether additional training or modifications of the technique may be warranted. This study tested the null hypothesis that clinician experience does not significantly affect the IOA outcomes.

## 2. Materials and Methods

### 2.1. Study Design and Ethical Approval

This retrospective observational study has been written according to the Preferred Reporting items for Observational studies in Endodontics (PROBE) 2023 guidelines. Ethical approval was obtained from the University Health Sciences Research Ethics Board (Protocol #46945; approved on 15 August 2024). All the patients provided verbal and written informed consent for the collection, use, and anonymous publication of their data.

### 2.2. Clinical Settings

This study was based on a chart review of two distinct clinical settings. The first was a university-based graduate endodontic clinic, where a single endodontic resident (AA) performed both the IOA and subsequent endodontic treatments or retreatment. Before joining the residency program, the resident had acquired 3 years of clinical experience as a general dentist, but lacked prior clinical experience, specifically in administering IOA, except for foundational training received during residency. This foundational training encompassed theoretical instruction, simulation-based practice using models, and practical training with the QuickSleeper 5 (QS5) system (Dental Hi Tec, Cholet Cedex, France).

The second setting was a private endodontic practice, where an experienced endodontist (JLG) performed the IOA and subsequent endodontic treatments or retreatments. JLG possessed 7 years of specialized clinical experience in endodontics, complemented by an additional 3 years of prior experience as a general dentist. JLG had routinely utilized the QS5 system for the previous 6 years, administering IOA approximately 8–10 times per week specifically for anesthetizing mandibular molars.

The data were collected from February 2023 to June 2024, corresponding to the period during which sufficient documentation was available for analysis.

### 2.3. Sample Size Determination

The sample size was estimated based on the null hypothesis that the clinician’s experience did not significantly affect the effectiveness of IOA. We assumed that the probability of achieving effective IOA in the endodontic resident group was 0.35, and in the endodontist group, this was 0.65. With a significance level of α = 0.05 and a power of β = 0.8, the calculated sample size was 44 patients. To account for the 20% dropout rate, the final sample size was 56 patients.

### 2.4. Participants

This study included 72 patients, 37 and 35 of whom were treated by an endodontic resident (AA) and an experienced endodontist (JLG), respectively. Each clinician administered IOA and performed the endodontic treatment or retreatment on their respective patients.

The patients were eligible if they were classified as an American Society of Anesthesiologists class I or II and required the endodontic treatment or retreatment of the permanent mandibular molars. The patients were excluded if they had an American Society of Anesthesiologists classification of III or higher; known allergic reactions to local anesthetics, such as lidocaine and sulfite preservatives; or had recently used analgesics, anti-inflammatories, or central nervous system depressants within 6 h before treatment. The additional exclusion criteria included the existence of infection or a lesion at the site of injection, the need for sedation, and the prior administration of local anesthesia in the same region within the last 4 days.

### 2.5. Pre-Anesthetic Assessment

Before IOA administration, all the patients rated their preoperative pain using a Visual Analog Scale (VAS) [38]. The VAS was classified into four levels: no pain (0 mm), mild pain (greater than 0 mm up to 54 mm), moderate pain (above 54 mm, but less than 114 mm), and severe pain (114 mm or more).

### 2.6. IOA Administration

The QS5 system was used for IOA administration following the manufacturer’s protocol. This electronically controlled injection device uses a rotating needle mechanism to perforate the cortical bone, allowing for direct anesthetic deposition into the cancellous bone. The system features electronically regulated flow control to ensure a consistent injection rate, reduce pressure-related discomfort, and improve anesthetic diffusion [39]. The QS5 has three flow settings: low, high, and intraosseous. The low and high settings are generally used for techniques such as inferior alveolar nerve block and infiltration. In this study, the intraosseous setting was used for all the steps, as recommended by the manufacturer.

IOA administration began with the insertion of a 27-gauge, 16 mm needle into the mucosa until it contacted bone, delivering 0.3–0.45 mL of 2% lidocaine with 1:100,000 epinephrine (Septodont, Saint-Maur-des-Fossés, France) for soft tissue anesthesia. Once the mucosa was anesthetized, the same needle, powered by a QS5 rotating mechanism, perforated the cortical bone, reaching the cancellous bone either mesial or distal to the target tooth, or in rare cases both. The remainder of the first carpule (1.35–1.5 mL) was injected into the cancellous bone.

A second carpule (1.8 mL) of 2% lidocaine with 1:100,000 epinephrine (Septodont, Saint-Maur-des-Fossés, France) was injected at the same perforation site without activating the rotation mechanism. If the site could not be relocated, the rotating needle was reactivated to create a perforation. Following IOA administration, but before endodontic treatment, the patients were instructed to indicate their pain level, which was recorded to assess the pain associated specifically with IOA administration.

### 2.7. Endodontic Treatment and Pain Assessment

Each clinician performed endodontic treatment or retreatment on the anesthetized patients. The patients were asked to lift their hands if they experienced discomfort during the procedure. A rubber dam was placed, and endodontic treatment was initiated. If a patient reported pain, the treatment was paused, the rubber dam was removed, and the patient reported their pain level with the VAS.

For the patients who experienced moderate or severe pain, further anesthesia was administered, which included IANB, buccal infiltration, periodontal ligament injection, or a second IOA. These cases were considered anesthesia failures and were excluded from further evaluation. If no pain was reported during the procedure, the patients were questioned about their experience at the end of the treatment. Anesthesia was considered successful if the patients experienced no pain or only mild discomfort during endodontic treatment or retreatment.

All the clinical data were extracted from electronic patient records by an external dentist (MA) between 16 August 2024 and 30 August 2024.

### 2.8. Statistical Analysis

Statistical analyses were performed using R 4.5.0 [40]. The significance level was *p* < 0.05. The patient pain levels during the operation were classified as either no/mild pain or moderate/severe pain and were recorded as binary variables. The clinicians’ experience was categorized as either residents or endodontists. The patients were assumed independent of each other. The patients with missing pain data were excluded from analysis.

To test the first null hypothesis that the probability of achieving no or mild pain was 0.5, we conducted a one-sided binomial test. To test the second null hypothesis that clinician experience does not affect IOA effectiveness, we compared the proportion of patients with no/mild pain between the resident and endodontist groups using a two-sample binomial test.

Chi-square tests were used to analyze the association between the patient- and tooth-level characteristics and pain level immediately after IOA, as well as IOA effectiveness. The patient-level characteristics included sex. The tooth-level characteristics included tooth type, pulpal diagnosis, periapical diagnosis, preoperative pain, IOA location, and the type of treatment provided. All the characteristics were treated as categorical variables.

An unadjusted logistic regression model was applied to evaluate the association between clinician experience and the odds of experiencing pain during dental procedures. Additionally, a confounder-adjusted logistic regression model was employed to adjust for clinically relevant confounders that might have affected pain during the procedure. The confounders included sex, preoperative pain, tooth type, pulpal diagnosis, and tooth location. For both the models, the estimated odds ratios and their corresponding confidence intervals are provided.

Power analysis was performed to determine the minimum sample size. The sample size was determined based on the expected probabilities of achieving profound anesthesia in the endodontic resident group and in the endodontist group. Since the exact values of both the probabilities are unknown, a range of plausible values were considered. We controlled the significant level at 0.05 and the power at 0.8.

## 3. Results

A total of 72 patients were included, of whom 37 were treated by a resident, and 35 by an endodontist, both of whom administered IOA and performed the subsequent endodontic procedures. Table 1 presents the distribution of demographic and clinical variables between the two groups, along with the *p*-values for statistical comparison.

Significant differences were observed in the tooth type (*p* = 0.009), pulpal diagnosis (*p* = 0.014), and IOA location (*p* = 0.045) between the two groups. The resident group treated a significantly higher proportion of mandibular first molars (78.4%), whereas the endodontist group treated more mandibular second molars (54.3%). Additionally, the pulpal diagnoses varied significantly between the groups, with necrotic cases comprising 51.4% of the endodontist group and 18.9% of the resident group, while the resident group treated a higher proportion of cases diagnosed with irreversible pulpitis (32.4%) and previously treated or initiated teeth (48.6%). Furthermore, the location of IOA placement varied, with the residents predominantly administering IOA at the distal site (86.5%), while the endodontist group had a higher proportion of mesial IOA (27.3%) and combined mesial and distal IOA (9.1%).

During IOA administration, 98.6% of all the patients reported no or only mild pain (97.2% in the endodontist group; 100% in the resident group), with no significant difference based on clinician experience (χ^2^ = 1.946, *p* = 0.378).

Profound anesthesia was achieved in 94.1% of the cases (96.8% in the endodontist group and 91.9% in the resident group), with no significant difference between the groups (*p* = 0.738).

Complications were rare, with two cases of perforator separation (5.4%), both occurring during IOA administration by the resident. However, statistical analysis demonstrated no significant association between clinician experience and the complication rate (*p* = 0.553).

## 4. Discussion

### 4.1. Clinical Significance of This Study

This study aimed to evaluate whether clinician experience influences the IOA outcomes and found that it had no significant impact on pain perception during IOA administration, anesthesia success, or the complication rates.

While prior research has assessed IOA effectiveness, it has not accounted for the clinician’s level of expertise. To our knowledge, this is the first study to explore the relationship between clinician experience and the IOA outcomes. By addressing this gap, this study provides a new insight into the consistency of IOA outcomes across different levels of clinical experience.

### 4.2. Methodology Considerations

This study considered success as experiencing no or mild pain during treatment, a criterion that aligns with those of the previous studies [30,41,42,43]. Many prior IOA studies have relied on electrical pulp testing (EPT) to assess anesthesia effectiveness [34,44]. However, EPT may not accurately reflect profound anesthesia during endodontic treatment, as patients who test negative for EPT may still experience pain [6]. By focusing on actual treatment pain rather than on the pulp testing responses, this study provides a clinically relevant assessment of IOA effectiveness.

A broader range of pulpal and periapical conditions was included, unlike the earlier studies that primarily focused on symptomatic irreversible pulpitis (SIP) and vital pulps [44]. SIP cases are more challenging to anesthetize [45]; however, endodontists and dentists frequently treat mandibular molars with various pulpal and periapical conditions. This broader inclusion improves the applicability of the study to real-world endodontic scenarios and supports the adoption of IOA as the primary anesthetic technique replacing IANB. The sample size was calculated based on the null hypothesis that the clinician’s experience does not significantly affect effectiveness.

Furthermore, this study examined IOA delivered using the QS5 system, whereas most previous IOA studies evaluated the Stabident and X-Tip systems (Dentsply Inc., York, PA, USA) [44]. Evaluating IOA using the QS5 provides valuable data for clinicians considering alternative IOA delivery systems.

### 4.3. Impact of Clinician Experience on the IOA Outcomes

The inclusion of inexperienced operators in clinical research is increasingly used to evaluate the usability, learning curve, and technique sensitivity of endodontic procedures. This approach offers insight into how novice clinicians interact with procedure-sensitive systems and informs educational strategies. One study assessed dental students’ perceptions of Ni-Ti rotary systems in a simulated setting, highlighting the value of operator experience in shaping training protocols [46]. The present study builds on this framework by examining IOA outcomes across varying experience levels.

**Figure 1:**
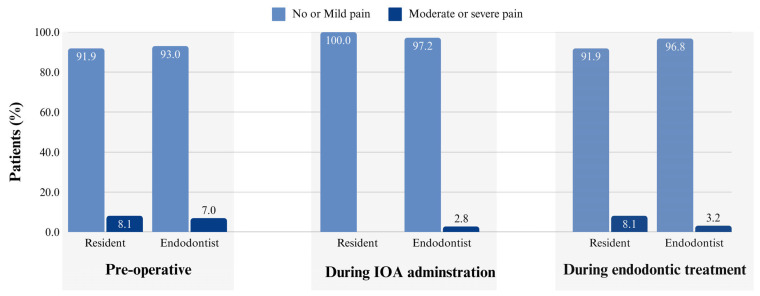
This bar graph presents the percentage of patients reporting no or mild pain versus moderate or severe pain at three key stages: preoperative, during intraosseous anesthesia (IOA) administration, and during endodontic treatment or retreatment. The data were compared between the residents and the endodontists to evaluate how the clinicians’ experience influenced pain perception.

This study found that clinician experience did not significantly affect the IOA outcomes, including pain during IOA administration, pain during endodontic treatment or retreatment, and the complication rates. Overall, 97.1% of the patients reported no or mild pain during IOA administration (Figure 1), which is consistent with the previous studies [4,6]. Although clinician experience did not play a significant role, preoperative pain emerged as a key influencing factor, with a one-level increase in the level of preoperative pain associated with a 1.32-fold increase in reported discomfort. This heightened pain response may be attributed to peripheral and central sensitization as well as patient anxiety [45].

Profound anesthesia was achieved in 94.1% of the cases across both the clinician groups, with no significant difference between the residents and the endodontists (Figure 1). Although concerns have been raised about the perceived complexity of IOA [37], this study demonstrated that clinicians with varying levels of experience can effectively administer the technique. The high success rate observed falls within the previously reported range of 89.3–96.8% for IOA effectiveness [20,21,27], further supporting its broader adoption in endodontic practice.

**Figure 2:**
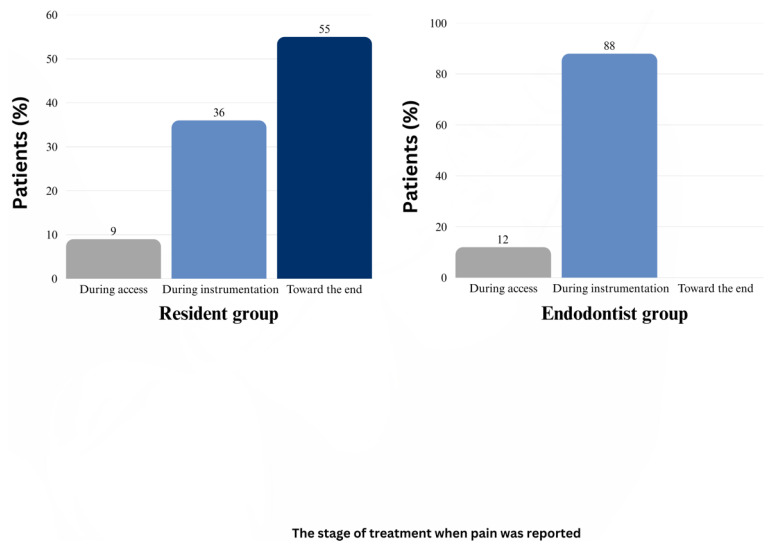
This bar graph illustrates the distribution of pain onset at the different stages of endodontic treatment in the patients treated by the residents and the endodontists. The stages analyzed included access preparation, instrumentation, and the end of the procedure. In the resident group, pain onset was most frequently reported toward the end of the procedure (55%), followed by instrumentation (36%) and access preparation (9%). In contrast, in the endodontist group, pain was most commonly reported during instrumentation (88%), with fewer cases occurring during access preparation (12%), and no patients reported pain toward the end of the procedure.

Notably, mild pain during treatment occurred more frequently in the resident group (51.4%) than in the endodontist group (17.1%) (Figure 2). Further analysis demonstrated that in the resident group, most pain incidents occurred toward the end of the procedure (after 1.5 h of treatment).

Previous research has also demonstrated that the depth of pulpal anesthesia declines as the treatment progresses. One study reported that for the first molar, the depth of pulpal anesthesia started to diminish 15 min after IOA administration, with only 66% to 74% of patients maintaining anesthesia for 30 min [47]. Another study found that the amount of IOA steadily declined over a 60 min period [34].

These findings indicate that the increased reports of pain during longer procedures were likely attributed to the natural waning of IOA over time, rather than clinician experience with IOA administration itself.

Complications were infrequent, with only two instances of perforator separation observed, both occurring in the resident group during their eighth and tenth IOA attempts. The first incident took place distal to the mandibular second molar in a patient who could not open their mouth wide. Restricted access may have contributed to the inadvertent bending of the perforator during rotation, ultimately leading to separation. Retrieval required flap elevation, after which the separated instrument was removed using a cotton plier. The patient experienced no postoperative pain or complications.

**Figure 3:**
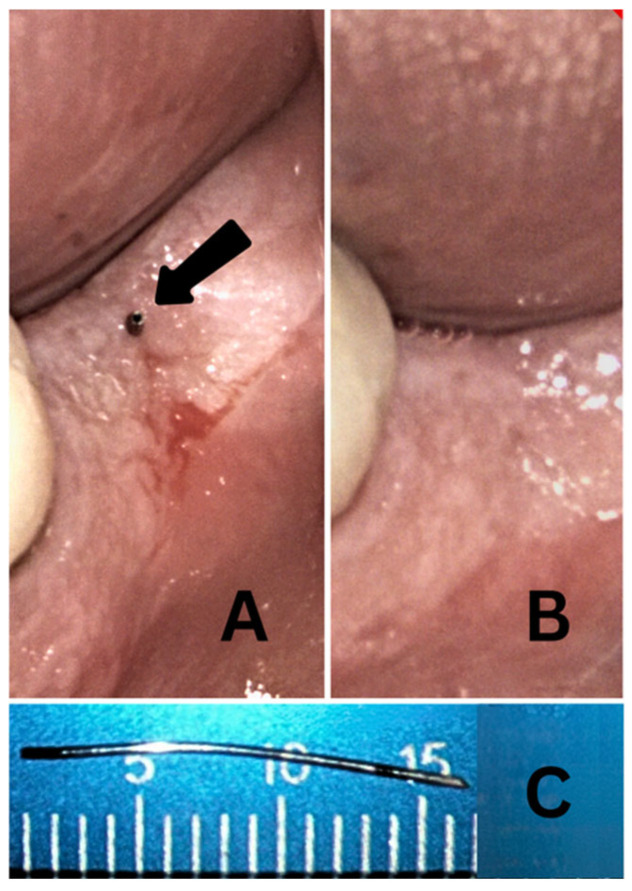
(**A**) A clinical photograph of a separated perforator (black arrow) in the retromolar pad area (resident group). (**B**) After the removal of the perforator with a needle holder. (**C**) The separated perforator.

The second perforator separation (Figure 3) occurred distal to the mandibular first molar, which was the terminal tooth in the arch. This may also have resulted from the bending of the perforator during insertion. In this case, a portion of the perforator remained visible above the gingival margin, allowing for the resident to retrieve it nonsurgically using a needle holder. No postoperative complications were reported.

It is possible that both these events were partially related to the straight handpiece design of the QS5 system, which may pose ergonomic limitations in posterior or restricted areas.

Statistical analysis demonstrated no significant association between clinician experience and the complication rates (*p* = 0.553), suggesting that these incidents were likely case-dependent, rather than directly related to experience. The overall low incidence of complications is consistent with the previous studies, reinforcing the safety of IOA [27,30].

Although experience level was not a determining factor, careful case selection may help minimize the risk of perforator separation, particularly in difficult-to-access areas such as the retromolar region. Additionally, alternative IOA delivery systems such as X-Tip, which employs a contra-angle handpiece, may provide better access in challenging cases and help further reduce risks.

### 4.4. Clinical Applications

These findings have important clinical implications, particularly for training and practice. The results suggest that IOA can be reliably administered by both residents and endodontists, reinforcing its utility as a predictable and effective anesthetic technique. This may encourage the broader adoption of IOA in endodontic training programs and routine clinical practice, particularly in cases where conventional IANB techniques are ineffective. Additionally, IOA’s reliability across different experience levels may help standardize its use, ensuring consistent patient outcomes regardless of clinician expertise.

### 4.5. Limitations and Future Directions

As a retrospective study, this research was inherently subject to selection bias and lacked the controlled conditions of a prospective randomized controlled trial. While the retrospective design allowed for the inclusion of a diverse patient population from university and private practice settings, it limited the control over potential confounding variables. To help reduce selection bias, strict inclusion and exclusion criteria were applied, and an independent evaluator performed data collection to ensure consistency across the clinical environments. Nevertheless, future studies should adopt randomized controlled designs, ideally stratifying the patients by clinician experience levels to better balance the baseline characteristics and minimize bias. Incorporating blinding procedures—such as blinding the evaluators or patients to the clinician’s experience—can further improve the objectivity of outcome assessments.

Another limitation is the possible influence of different treatment settings on patient-reported pain. Patients seen in private practices may have higher expectations than those treated in academic settings, potentially affecting their perception and reporting of pain. This study used validated pain assessment tools (e.g., VAS) with standardized instructions and consistent timing to reduce the variability across settings. However, future studies could strengthen this approach by blinding patients to the clinician’s experience level and ensuring uniform pretreatment communication to standardize expectations.

Additionally, the differences in clinical proficiency between the residents and the endodontists may have influenced pain reporting. This study included only one resident and one endodontist, limiting the generalizability of the results. Future research should include multiple clinicians with similar endodontic experience, but varying levels of expertise with IOA. This would help isolate the effect of IOA-related experience from general clinical skills. Stratifying the clinicians based on the number of IOA procedures previously performed would also allow for more meaningful subgroup analysis and improve external validity.

Patient-related factors, such as baseline anxiety, anatomical variations, and a history of anesthetic failure, may also influence pain perception and anesthetic success. Future studies should assess these variables using standardized tools—for example, the Modified Dental Anxiety Scale for anxiety—and document relevant anatomical features such as cortical bone thickness. Including these factors as covariates in multivariate analyses could help clarify the independent effect of clinician experience on the anesthetic outcomes.

Finally, larger sample sizes and multi-center trials involving various geographic and clinical contexts would enhance both the statistical power and generalizability of findings. By addressing these limitations through improved study design—such as randomization, blinding, and broader clinician and patient inclusion—future research can more precisely evaluate how clinician experience influences the anesthesia outcomes in endodontic practice.

## 5. Conclusions

Within the limitations of this study, the null hypothesis was accepted, as clinician experience did not significantly affect the IOA outcomes, including effectiveness, patient comfort, and safety. These findings suggest that the IOA can be used effectively by clinicians with different levels of experience, supporting its broader implementation in endodontic practice. Furthermore, the results underscore the potential to incorporate IOA into standardized training programs, promoting its safe and effective use from the early stages of clinical education and informing guidelines for broader, evidence-based implementation.

## Figures and Tables

**Table 1:** Distribution of patient variables between residents and endodontists. This table summarizes patient demographics and clinical variables (sex, tooth type, pulpal diagnosis, preoperative pain, and location of IOA administration) with associated *p*-values, indicating statistical significance between groups.

Variable	Category	Resident (n = 37)	Expert (n = 35)	*p*-Value
Sex	Female	21 (56.8%)	14 (40.0%)	0.236
	Male	16 (43.2%)	21 (60.0%)	
Tooth Type	Mandibular 1st molar	29 (78.4%)	16 (45.7%)	0.009
	Mandibular 2nd molar	8 (21.6%)	19 (54.3%)	
Pulpal Diagnosis	Irreversible Pulpitis	12 (32.4%)	8 (22.9%)	0.014
	Necrosis	7 (18.9%)	18 (51.4%)	
	Previously treated/initiated	18 (48.6%)	9 (25.7%)	
Preoperative Pain	None	27 (73.0%)	20 (69.0%)	0.938
	Mild	7 (18.9%)	7 (24.1%)	
	Moderate	2 (5.4%)	1 (3.4%)	
	Severe	1 (2.7%)	1 (3.8%)	
Location of IOA	Distal	32 (86.5%)	21 (63.6%)	0.045
	Mesial	5 (13.5%)	9 (27.3%)	
	Mesial + Distal	0 (0.0%)	3 (9.1%)	

## Data Availability

Available upon request.

## References

[1] Olch P.D., William S. (1975). Halsted and local anesthesia: Contributions and complications. Anesthesiology.

[2] Claffey E., Reader A., Nusstein J., Beck M., Weaver J. (2004). Anesthetic efficacy of articaine for inferior alveolar nerve blocks in patients with irreversible pulpitis. J. Endod..

[3] Zanjir M., Lighvan N.L., Yarascavitch C., Beyene J., Shah P.S., Azarpazhooh A. (2019). Efficacy and safety of pulpal anesthesia strategies during endodontic treatment of permanent mandibular molars with symptomatic irreversible pulpitis: A systematic review and network meta-analysis. J. Endod..

[4] Dunbar D., Reader A., Nist R., Beck M., Meyers W.J. (1996). Anesthetic efficacy of the intraosseous injection after an inferior alveolar nerve block. J. Endod..

[5] Childers M., Reader A., Nist R., Beck M., Meyers W.J. (1996). Anesthetic efficacy of the periodontal ligament injection after an inferior alveolar nerve block. J. Endod..

[6] Nusstein J., Reader A., Nist R., Beck M., Meyers W.J. (1998). Anesthetic efficacy of the supplemental intraosseous injection of 2% lidocaine with 1:100,000 epinephrine in irreversible pulpitis. J. Endod..

[7] Reisman D., Reader A., Nist R., Beck M., Weaver J. (1997). Anesthetic efficacy of the supplemental intraosseous injection of 3% mepivacaine in irreversible pulpitis. Oral Surg. Oral Med. Oral Pathol. Oral Radiol. Endod..

[8] Cohen H.P., Cha B.Y., Spångberg L.S. (1993). Endodontic anesthesia in mandibular molars: A clinical study. J. Endod..

[9] Nusstein J., Claffey E., Reader A., Beck M., Weaver J. (2005). Anesthetic effectiveness of the supplemental intraligamentary injection, administered with a computer-controlled local anesthetic delivery system, in patients with irreversible pulpitis. J. Endod..

[10] Fowler S., Drum M., Reader A., Beck M. (2016). Anesthetic success of an inferior alveolar nerve block and supplemental articaine buccal infiltration for molars and premolars in patients with symptomatic irreversible pulpitis. J. Endod..

[11] Matthews R., Drum M., Reader A., Nusstein J., Beck M. (2009). Articaine for supplemental buccal mandibular infiltration anesthesia in patients with irreversible pulpitis when the inferior alveolar nerve block fails. J. Endod..

[12] Gow-Gates G.A. (1973). Mandibular conduction anesthesia: A new technique using extraoral landmarks. Oral Surg. Oral Med. Oral Pathol..

[13] Akinosi J.O. (1977). A new approach to the mandibular nerve block. Br. J. Oral Surg..

[14] Click V., Drum M., Reader A., Nusstein J., Beck M. (2015). Evaluation of the Gow-Gates and Vazirani-Akinosi techniques in patients with symptomatic irreversible pulpitis: A prospective randomized study. J. Endod..

[15] Goldberg S., Reader A., Drum M., Nusstein J., Beck M. (2008). Comparison of the anesthetic efficacy of the conventional inferior alveolar, Gow-Gates, and Vazirani-Akinosi techniques. J. Endod..

[16] Masselink B.H. (1910). The advent of painless dentistry. Dent. Cosm..

[17] Lilienthal B. (1975). A clinical appraisal of intraosseous dental anesthesia. Oral Surg. Oral Med. Oral Pathol..

[18] Parente S.A., Anderson R.W., Herman W.W., Kimbrough W.F., Weller R.N. (1998). Anesthetic efficacy of the supplemental intraosseous injection for teeth with irreversible pulpitis. J. Endod..

[19] Reitz J., Reader A., Nist R., Beck M., Meyers W.J. (1998). Anesthetic efficacy of the intraosseous injection of 0.9 mL of 2% lidocaine (1:100,000 epinephrine) to augment an inferior alveolar nerve block. Oral Surg. Oral Med. Oral Pathol. Oral Radiol..

[20] Gaudin A., Clouet R., Boëffard C., Laham A., Martin H., Amador Del Valle G., Enkel B., Prud’homme T. (2023). Comparing intraosseous computerized anaesthesia with inferior alveolar nerve block in the treatment of symptomatic irreversible pulpitis: A randomized controlled trial. Int. Endod. J..

[21] Simeonova E., Tsanova S., Zagorchev P. (2020). Effectiveness of primary intraosseous anesthesia in the endodontic treatment of mandibular molars with irreversible pulpitis. J. IMAB.

[22] Farhad A., Razavian H., Shafiee M. (2018). Effect of intraosseous injection versus inferior alveolar nerve block as primary pulpal anaesthesia of mandibular posterior teeth with symptomatic irreversible pulpitis: A prospective randomized clinical trial. Acta Odontol. Scand..

[23] Demir E., Ataoglu H. (2020). Clinical evaluation of efficacy of transcortical anesthesia for the extraction of impacted mandibular third molars: A randomized controlled trial. J. Dent. Anesth. Pain Med..

[24] Jain S.D., Carrico C.K., Bermanis I., Rehil S. (2020). Intraosseous anesthesia using dynamic navigation technology. J. Endod..

[25] Sovatdy S., Vorakulpipat C., Kiattavorncharoen S., Saengsirinavin C., Wongsirichat N. (2018). Inferior alveolar nerve block by intraosseous injection with Quicksleeper^®^ at the retromolar area in mandibular third molar surgery. J. Dent. Anesth. Pain Med..

[26] Vatankhah M., Dianat O., Zargar N., Bejestani S.G., Baghban A.A., Shojaeian S. (2024). Efficacy of QuickSleeper Intraosseous Injection of 4% Articaine in Mandibular First Molars With Symptomatic Irreversible Pulpitis: A Randomized Controlled Trial. Anesth. Prog..

[27] Pereira L.A., Groppo F.C., Bergamaschi Cde C., Meechan J.G., Ramacciato J.C., Motta R.H., Ranali J. (2013). Articaine (4%) with epinephrine (1:100,000 or 1:200,000) in intraosseous injections in symptomatic irreversible pulpitis of mandibular molars: Anesthetic efficacy and cardiovascular effects. Oral Surg. Oral Med. Oral Pathol. Oral Radiol..

[28] Guglielmo A., Reader A., Nist R., Beck M., Weaver J. (1999). Anesthetic efficacy and heart rate effects of the supplemental intraosseous injection of 2% mepivacaine with 1:20,000 levonordefrin. Oral Surg. Oral Med. Oral Pathol. Oral Radiol. Endod..

[29] Replogle K., Reader A., Nist R., Beck M., Weaver J., Meyers W.J. (1999). Cardiovascular effects of intraosseous injections of 2 percent lidocaine with 1:100,000 epinephrine and 3 percent mepivacaine. J. Am. Dent. Assoc..

[30] Nusstein J., Kennedy S., Reader A., Beck M., Weaver J. (2003). Anesthetic efficacy of the supplemental X-tip intraosseous injection in patients with irreversible pulpitis. J. Endod..

[31] Pol R., Ruggiero T., Bezzi M., Camisassa D., Carossa S. (2022). Programmed-release intraosseous anesthesia as an alternative to lower alveolar nerve block in lower third molar extraction: A randomized clinical trial. J. Dent. Anesth. Pain. Med..

[32] Saber S., Hashem A.A., Khalil D.M., Pirani C., Ordinola-Zapata R. (2021). Efficacy of four local anaesthesia protocols for mandibular first molars with symptomatic irreversible pulpitis: A randomized clinical trial. Int. Endod. J..

[33] Reitz J., Reader A., Nist R., Beck M., Meyers W.J. (1998). Anesthetic efficacy of a repeated intraosseous injection given 30 min following an inferior alveolar nerve block/intraosseous injection. Anesth. Prog..

[34] Gallatin J., Reader A., Nusstein J., Beck M., Weaver J. (2003). A comparison of two intraosseous anesthetic techniques in mandibular posterior teeth. J. Am. Dent. Assoc..

[35] Coggins R., Reader A., Nist R., Beck M., Meyers W.J. (1996). Anesthetic efficacy of the intraosseous injection in maxillary and mandibular teeth. Oral Surg. Oral Med. Oral Pathol. Oral Radiol. Endod..

[36] Woodmansey K.F., White R.K., He J. (2009). Osteonecrosis related to intraosseous anesthesia: Report of a case. J. Endod..

[37] Bangerter C., Mines P., Sweet M. (2009). The use of intraosseous anesthesia among endodontists: Results of a questionnaire. J. Endod..

[38] Heft M.W., Parker S.R. (1984). An experimental basis for revising the graphic rating scale for pain. Pain.

[39] Dental Hi Tec. https://www.dentalhitec.com.

[40] R Core Team (2024). R: A Language and Environment for Statistical Computing.

[41] Bigby J., Reader A., Nusstein J., Beck M., Weaver J. (2006). Articaine for supplemental intraosseous anesthesia in patients with irreversible pulpitis. J. Endod..

[42] Bhuyan A.C., Latha S.S., Jain S., Kataki R. (2014). Anesthetic efficacy of the supplemental X-tip intraosseous injection using 4% articaine with 1:100,000 adrenaline in patients with irreversible pulpitis: An in vivo study. J. Conserv. Dent..

[43] Idris M., Sakkir N., Naik K.G., Jayaram N.K. (2014). Intraosseous injection as an adjunct to conventional local anesthetic techniques: A clinical study. J. Conserv. Dent..

[44] Replogle K., Reader A., Nist R., Beck M., Weaver J., Meyers W.J. (1997). Anesthetic efficacy of the intraosseous injection of 2% lidocaine (1:100,000 epinephrine) and 3% mepivacaine in mandibular first molars. Oral Surg. Oral Med. Oral Pathol. Oral Radiol. Endod..

[45] Hargreaves K.M., Keiser K. (2002). Local anesthetic failure in endodontics: Mechanisms and management. Endod. Top..

[46] Puleio F., Tosco V., Monterubbianesi R., Pirri R., Alibrandi A., Pulvirenti D., Simeone M. (2025). Comparison of Four Ni-Ti Rotary Systems: Dental Students’ Perceptions in a Multi-Center Simulated Study. Dent. J..

[47] Jensen J., Nusstein J., Drum M., Reader A., Beck M. (2008). Anesthetic efficacy of a repeated intraosseous injection following a primary intraosseous injection. J. Endod..

